# A study of the genus *Paraputo* Laing, 1929 of China, with description of two new species (Hemiptera, Sternorrhyncha, Coccomorpha)

**DOI:** 10.3897/zookeys.709.15161

**Published:** 2017-10-18

**Authors:** Jiang-Tao Zhang, San-An Wu

**Affiliations:** 1 Key Laboratory for Silviculture and Conservation of Ministry of Education, Beijing Forestry University, Beijing, China

**Keywords:** Mealybugs, new combination, Pseudococcidae

## Abstract

A study of the genus *Paraputo* Laing, 1929 (Coccomorpha: Pseudococcidae) from China is provided. Eight *Paraputo* species are recognized in China, of which two species, *P. platani* **sp. n.** and *P. yunnanensis* **sp. n.**, are described as new and *P. banzigeri* Williams, 2004 is recorded from China for the first time. Two new combinations are introduced, involving transfer of *P. citricola* Tang, 1992 and *P. sinensis* Borchsenius, 1962 to the genus *Formicococcus* Takahashi, 1928. A key to the *Paraputo* species of China is provided.

## Introduction

The genus *Paraputo* was established by Laing (1929) with *P. ritchiei* Laing as its type species, which was described from Tanzania in the Afrotropical Region. Ferris (1955) erected the genus *Cataenococcus* with *Dactylopius olivaceus* Cockerell as its type species, later listed as a part of *Paraputo* by Tang (1992). Borchsenius (1960) described the genus *Lachnodiopsis* with *L. szemaoensis* Borchsenius as its type species, which was synonymized with *Paraputo* by Williams (2004) based on the study of original material. Currently, the genus *Paraputo* has been widely studied in many geographic regions: De Lotto (1964) and Millar (2002) in the African region; McKenzie (1967), Miller and McKenzie (1971, 1973), Williams and Granara de Willink (1992) in the American region; Williams and Watson (1988), Williams (2004, 2005) in the Pacific and southern Asia regions; Tang (1992) and Wang (2001) in the Chinese region. Currently, the genus includes 81 species worldwide (Ben-Dov and Williams 2006, García Morales et al. 2017).

The genus *Paraputo* is morphologically similar to *Formicococcus*; both have body of adult female broadly oval to rotund, legs stout, ostioles prominent, and cerarii rich containing multiple conical setae. Williams (2004) reviewed these two genera, using the presence or absence of an anal lobe bar for generic separation, with the bar being absent in *Paraputo*. However, Danzig and Gavrilov-Zimin (2015) considered the bar as subject to individual variation, and separated *Paraputo* and *Formicococcus* according to the number of setae in anal ring. Herein, we have adopted the suggestion of Williams (2004), who regarded the number of ring setae as not having any generic significance; based on our studies, the anal ring usually bears six basic setae, and when more setae are present, the extra setae are usually slender and short and vary in their positions.

*Paraputo comantis* Wang and *P. gasteris* Wang were previously transferred to *Formicococcus* as *F. comantis* (Wang) and *F. gasteris* (Wang) by Tang (1992) and Wu and Zheng (2001) respectively, on account of each species possessing multiple setae in the anal ring. In this study, this character is not regarded as having generic significance (as in the statement above), so we still place these two species in *Paraputo* in view of the absence of any anal lobe bar. *Paraputo citricola* Tang and *P. sinensis* Borchsenius, in which an anal lobe bar is present on each anal lobe, are here transferred to *Formicococcus* as *F. citricola* (Tang) comb. n. and *F. sinensis* (Borchsenius) comb. n. With the two new species described herein, *Paraputo platani* sp. n. and *P. yunnanensis* sp. n., and the new Chinese record of *P. banzigeri* Williams, there are now eight *Paraputo* species in China: *P. albizzicola* Borchsenius, *P. banzigeri*, *P. comantis*, *P. gasteris*, *P. platani* sp. n., *P. porosus* Borchsenius, *P. szemaoensis* (Borchsenius) and *P. yunnanensis* sp. n. A key to the *Paraputo* species found in China is provided below.

## Materials and methods

The mealybug specimens were collected individually. The specimens were prepared using the method of Borchsenius (1950) and were mounted in Euparal. The terminology used follows that of Williams (2004). Measurements were made using a phase-contrast microscope (Leica DME) fitted with an ocular micrometer. Measurements are given in micrometers (μm) except for the length and width of the body, which are given in millimeters (mm); all measurements are given as minimum and maximum values. The drawings are arranged in the usual way for illustrating Coccomorpha, with the central drawing showing a slide-mounted specimen and the distribution of morphological features, and the enlarged drawings (not to scale) showing the detailed structure of important characters. Scale insect illustrations show the dorsal surface on the left side and the ventral surface on the right side.

The holotypes and paratypes of the new species are deposited in the Insect Collection, the Department of Forestry Protection, Beijing Forestry University, Beijing, China (**BFUC**).

## Taxonomy

### Genus Paraputo

Taxon classificationAnimaliaHemipteraPseudococcidae

Laing, 1929

Paraputo Laing, 1929: 473; Ferris 1955: 5; Williams 1958: 217, 1960: 419, 2004: 484, 2005: 3343; Morrison and Morrison 1966: 146; Matile-Ferrero 1978: 39; Williams and Watson 1988: 151; Tang 1992: 304; Ben-Dov 1994: 282; Danzig and Gavrilov-Zimin 2015: 27. Type species Paraputo ritchiei Laing, 1929, by original designation and monotypy (= Ripersia anomala Newstead, 1908).
Cataenococcus Ferris, 1955: 3; Williams 1960: 419; Williams and Granara de Willink 1992: 73; Ben-Dov 1994. Type species Dactylopius olivaceus Cockerell, by original designation. Synonymised by Tang 1992: 304.
Lachnodiopsis Borchsenius, 1960: 923; Tang 1992: 297; Wang 2001: 118; Williams 2004: 485. Type species Lachnodiopsis szemaoensis Borchsenius, by original designation. Synonymised by Williams 2004: 484.

#### Diagnosis.

Body of adult female broadly oval to rotund. Antennae 6 to 8 segmented. Legs well developed, stout, tibia + tarsus usually shorter than trochanter + femur; translucent pores normally present on hind coxae; claw stout, without a denticle. Anal ring generally situated at least its own length from apex of abdomen, bearing 6 or multiple setae. Circulus present or absent. Cerarii numbering 5–18 pairs; cerarii on posterior abdominal segments (including anal lobe cerarii) usually each containing multiple conical setae; sometimes intermediate cerarii or intermediate conical setae present. Ostioles well developed, with inner edges of lips sclerotized. Multilocular disc pores present, rarely absent. Oral collar tubular ducts present, usually across medial area and sometimes in marginal groups. Anal lobes ventrally membranous or with various degrees of sclerotization, never with an anal lobe bar. Dorsal setae usually minute and stiff, ventral surface usually with normal flagellate setae (adapted from Williams 2004).

### Paraputo albizzicola

Taxon classificationAnimaliaHemipteraPseudococcidae

Borchsenius, 1962

Paraputo albizzicola Borchsenius, 1962: 228; Tang 1992: 306; Wang 2001: 123–124; Danzig and Gavrilov-Zimin 2015: 31.

#### Host plant.

Fabaceae: *Albizzia lebbek*.

#### Distribution.

China (Yunnan).

#### Remark.

This species was described from one female, deposited in the Institute of Zoology, Chinese Academy of Sciences, Beijing, China (IZCAS) and probably now lost (Danzig and Gavrilov-Zimin 2015). We have therefor adopted the original combination of Borchsenius (1962) and are treating it as a valid species at present.

### Paraputo banzigeri

Taxon classificationAnimaliaHemipteraPseudococcidae

Williams, 2004

Paraputo banzigeri Williams, 2004: 497.

#### Material examined.

Three adult females, China: Yunnan, Puer city, Lancang Lahu Autonomous Country, on roots of *Cinnamomum japonicum* (Lauraceae) attended by ants, 18.x.2016, coll. Xu-bo Wang and Yao-guang Qin. Two adult females, China: Yunnan, Jinghong city, on roots of *Ficus microcarpa* (Moraceae), inside theca of the fungus *Phlebopus portentosus*, 22.vii.2012, coll. Jing Zhao. Three adult females, China: Yunnan, Xishuangbanna Dai Autonomous Prefecture, on *Ficus* sp. (Moraceae), ix.2009, coll. Yi-wei Fang.

#### Host plants.

Lauraceae: *Cinnamomum japonicum*; Moraceae: *Ficus* sp., *F. microcarpa*; Sapindaceae: *Dimocarpus longan*.

#### Distribution.

China (Yunnan), Thailand.

#### Biology.

This species was collected from *Cinnamomum japonicum* attended by ants, and sometimes inside the theca of the fungus *Phlebopus portentosus* on the roots of *Ficus microcarpa* (Zhang et al. 2015).

#### Remarks.

The material examined agrees with the original description by Williams (2004) except that there are a few oral collar tubular ducts present lateral to each first coxa. Good description and illustration are given by Williams (2004).

### Paraputo comantis

Taxon classificationAnimaliaHemipteraPseudococcidae

Wang, 1978

Paraputo comantis Wang, 1978: 416, 1982: 70–71.
Formicococcus comantis (Wang): Tang 1992: 288; Wang 2001: 113–114; Wu 2001: 201; Danzig and Gavrilov-Zimin 2015: 20.

#### Host plant.

Oleaceae: *Fraxinus chinensis*.

#### Distribution.

China (Zhejiang).

#### Remarks.

Based on the original description and illustration of Wang (1978), the venter of each anal lobe does not have an anal lobe bar; hence, this species is transferred back to *Paraputo*, as originally described.

### Paraputo gasteris

Taxon classificationAnimaliaHemipteraPseudococcidae

Wang, 1982

Paraputo gasteris Wang, 1982: 317.
Formicococcus gastrodiae Tang, 1992: 594.
Formicococcus gasteris (Wang): Wu and Zheng 2001: 201; Danzig and Gavrilov-Zimin 2015: 21.

#### Material examined.

Four adult females, China: Sichuan, Chengdu city, Sichuan University, under bark crack of *Platanus* sp. (Platanaceae), attended by ants, 18.vii.2016, coll. Ge Li and San-an Wu. Six adult females, China: Sichuan, Langzhong city, under bark crack of *Platanus* sp. (Platanaceae), attended by ants, 2.viii.2014, coll. Jiang-tao Zhang and Xu-bo Wang. Nine adult females, China: Guizhou, Guiyang city, Huaxi qu, in ants’ nest on *Celtis* sp. (Ulmaceae), 19.viii.2010, coll. San-an Wu and Yuan Lu. Two adult females, China: Guizhou, Guiyang city, Huaxi qu, in ants’ nest on *Populus* sp. (Salicaceae), 19.viii.2010, coll. San-an Wu and Yuan Lu.

#### Host plants.

Orchidaceae: *Gastrodia elata*; Platanaceae: *Platanus* sp.; Salicaceae: *Populus* sp.; Ulmaceae: *Celtis* sp.

#### Distribution.

China (Guizhou, Shaanxi, Sichuan).

#### Remarks.

Based on the sclerotization pattern of the ventral surface of each anal lobe (not forming an anal lobe bar), this species is transferred back to *Paraputo*, as *P. gasteris* Wang.

### Paraputo platani sp. n.

Taxon classificationAnimaliaHemipteraPseudococcidae

http://zoobank.org/12B08267-A54F-4C08-A6BF-16AAB593C9D0

#### Material examined.

*Holotype*. Adult female. China: Sichuan, Yaan city, Zhangjiashan Park, under bark crack of *Platanus* sp. (Platanaceae), attended by ants, 28.vii.2014, coll. Jiang-tao Zhang and Xu-bo Wang. *Paratypes*. Four adult females, same date and locality as holotype.

#### Other material examined.

Two adult females, China: Sichuan, Chongzhou city, Huaiyuan town, under bark crack of *Platanus* sp. (Platanaceae), attended by ants, 14.vii.2016, coll. Ge Li and San-an Wu.

#### Description.

**Figure 1.**
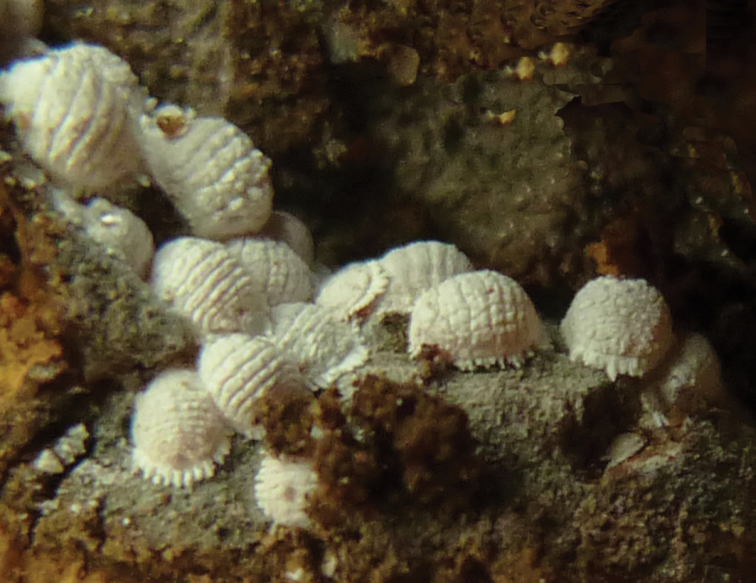
Habitus photo of *Paraputo platani* sp. n. on *Platanus* sp.

**Figure 2.**
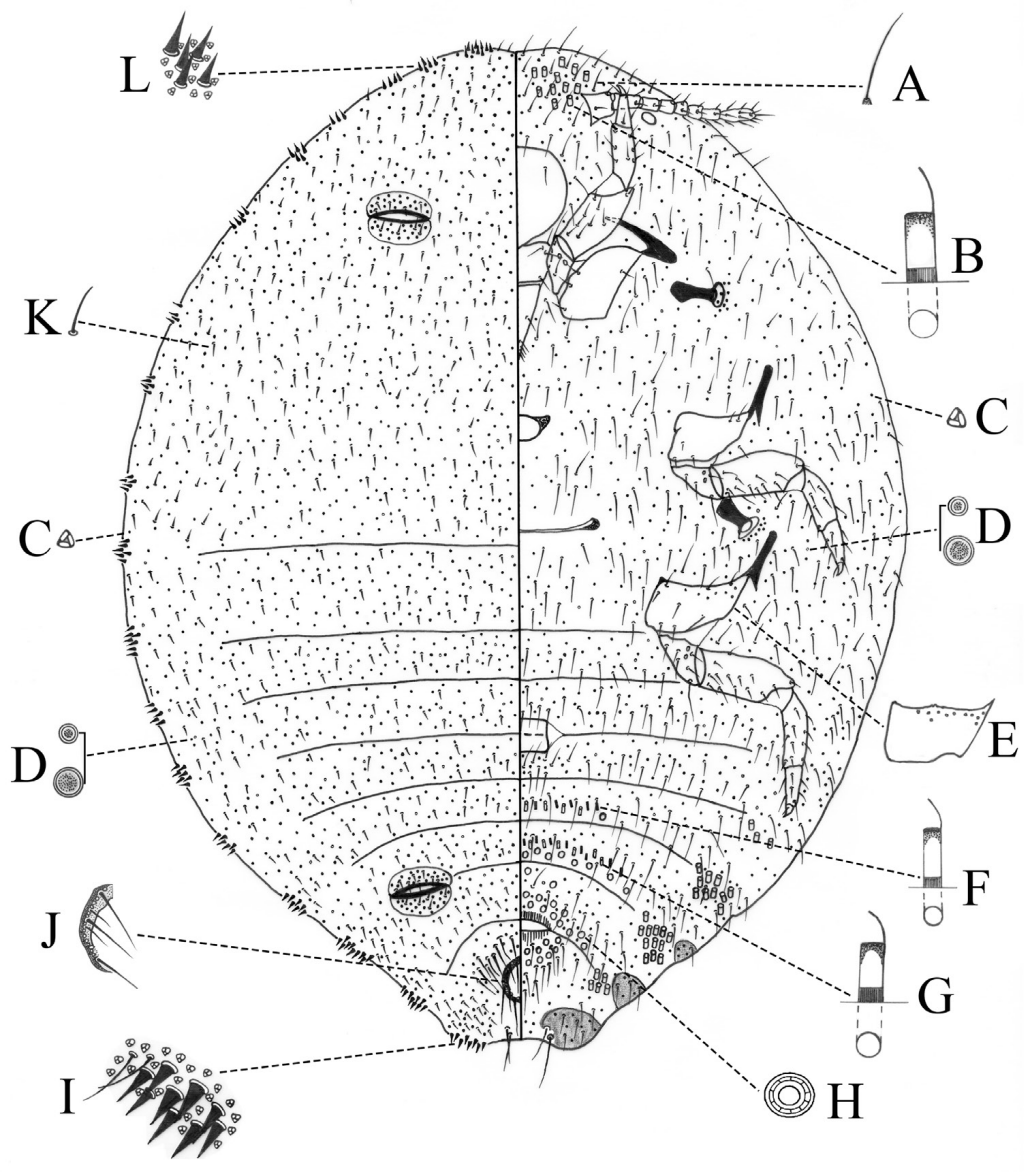
Adult female of *Paraputo platani* sp. n. **A** Ventral flagellate seta on head **B** Large type of oral collar tubular duct **C** Trilocular pore **D** Discal pore **E** Hind coxa **F** Small type of oral collar tubular duct **G** Intermediate type of oral collar tubular duct **H** Multilocular disc pore **I** Anal lobe cerarius **J** Anal ring **K** Dorsal seta **L** Cerarius on head.

In life (Fig. 1), adult female convex, segmentation prominent, coated with dense white mealy wax and with short dense white wax filaments around body margin. Body of adult female on microscope slide (Fig. 2) broadly oval, almost circular, 2.2–2.45 mm long and 1.85–2.05 mm wide. Anal lobes slightly prominent, ventral surface of each lobe with an apical seta 99.5–105.5 μm long, ratio of lengths of apical setae to anal ring setae 1: 0.92–1.02, with large sclerotized area occupying most of lobe. Other sclerotized areas also present on ventral margins on each side of abdominal segments VI and VII.

*Dorsum*. Ostioles well-developed, inner edges of lips sclerotized and each lip with 5–11 setae and numerous trilocular pores. Cerarii numbering 18 pairs. Anal lobe cerarii (C_18_) each containing 8–10 conical setae of different sizes, large setae each 23–29 μm long and 11.5–12 μm wide at base, situated with a group of trilocular pores and 2–4 setae on a membranous area. Penultimate cerarii (C_17_) and antepenultimate cerarii (C_16_) each containing 8–13 conical setae; C_14_ and C_15_ each containing 7–9 conical setae; frontal cerarii (C_1_) each containing 7–9 conical setae; other cerarii, each containing 3–8 conical setae. Anal ring 100–112.5 μm long and 80.5–91 μm wide, situated at nearly its own length from apex of abdomen, with two rows of pores, six long setae and 3–5 short setae, long seta each 78.5–107.5 μm long, shorter than anal ring length. Trilocular pores numerous, each 3–4 μm wide, evenly distributed. Multilocular disc pores and oral collar tubular ducts absent. Cisanal and obanal setae present, stout, 87–105.5 μm long. Dorsal setae short, each 19–28.5 μm long on head and 20–33.5 μm long on abdomen. Long flagellate setae present flanking anal ring, each 70–87 μm long. Discoidal pores of 2 sizes present: a large type, each slightly larger than or as wide as a trilocular pore; and a small type, each smaller than a trilocular pore; scattered.

*Venter*. Antennae each 357.5–390.5 μm long, 8-segmented (sometimes segment IV and V combined together); apical segment longest, bearing four fleshy setae. Eye spot oval, located at body margin posterior to antennal base. Legs well developed, stout; hind coxa wider than long, 108–146 μm long; hind trochanter + femur 316–366 μm long, hind tibia + tarsus 264–290 μm long; claw stout, 42–49 μm long, without denticle, claw digitules knobbed, each as long as claw. Ratio of lengths of hind tibia + tarsus to hind trochanter + femur 1: 1.18–1.26. Ratio of lengths of hind tibia to tarsus 1: 0.59–0.7. Translucent pores present on posterior surface of hind coxa. Clypeolabral shield 229–281 μm long. Labium 270–324 μm long. Ratio of lengths of labium to clypeolabral shield 1: 0.81–0.93. Circulus present, nearly square, 75–116 μm long and 125.5–150 μm wide, situated between abdominal segments III and IV, divided by intersegmental line. Trilocular pores evenly distributed, fewer than those on dorsum. Multilocular disc pores, each 9–9.5 μm in diameter, present posterior to vulva and on abdominal segments VI and VII, sometimes occurring on abdominal segment V. Oral collar tubular ducts of 3 sizes: large type, each 13–14 μm long and 5–6 μm wide, forming groups on margins of posterior abdominal segments, segment III with 1–2 or absent, segment IV with 3–9, segment V with 7–14, segment VI with 5–13, segment VII with 4–8 and 16–18 between antennal bases; intermediate type, each 11–12 μm long and 4–5 μm wide, present across posterior medial area of abdominal segments V–VI, sometimes present at inner edges of marginal groups of large ducts on abdominal segments VI and VII; small type, each 9–11 μm long and 3–4 μm wide, distributed across abdominal segments V–VI. Ventral setae slender, each 36–67 μm long on head and 35–57 μm on abdomen. Discoidal pores, same as those on dorsum, scattered.

#### Host plant.

Platanaceae: *Platanus* sp.

#### Distribution.

China (Sichuan).

**Biology.** This mealybug is found under bark crack of *Platanus* and is attended by ants.

#### Etymology.

The specific epithet is based on the Latin genitive of the host-plant name.

#### Remarks.

*Paraputo platani* sp. n. is most similar to *P. comantis* Wang in the number of cerarii, long setae present flanking anal ring and anal ring bearing more than six setae. However, *P. platani* sp. n. differs from *P. comantis* Wang by the following features (condition of *P. comantis* Wang given in parentheses): (i) posteriormost three cerarii (C_16_, C_17_ and C_18_) situated on membranous plates (those cerarii situated on sclerotized areas); (ii) translucent pores present on hind coxa (absent from hind coxa); (iii) oral collar tubular ducts distributed across abdominal segments V–VI (ducts distributed across segments V–VII).

### Paraputo porosus

Taxon classificationAnimaliaHemipteraPseudococcidae

Borchsenius, 1962

Paraputo porosus Borchsenius, 1962: 226; Tang 1992: 311; Wang 2001: 122–123; Danzig and Gavrilov-Zimin 2015: 43.

#### Material examined.

Three adult females, China: Yunnan, Kunming city, on *Robinia pseudacacia* (Fabaceae), 23.iv.1957, coll. N.S. Borchsenius.

#### Host plant.

Fabaceae: *Robinia pseudacacia*.

#### Distribution.

China (Yunnan).

#### Remarks.

In the original material studied, the ventral surface of each anal lobe is membranous, so *P. porosus* Borchsenius is still included in *Paraputo*.

### Paraputo szemaoensis

Taxon classificationAnimaliaHemipteraPseudococcidae

(Borchsenius, 1960)

Lachnodiopsis szemaoensis Borchsenius, 1960: 923; Wang 1982: 64, 2001: 120; Tang 1992: 298.
Paraputo szemaoensis (Borchsenius): Williams 2004: 484.
Formicococcus szemaoensis (Borchsenius): Danzig and Gavrilov-Zimin 2015: 26.

#### Material examined.

Two adult females, China: Yunnan, Puer city, Simao qu, on *Pasania* sp. (Fagaceae), 26.iii.1957, coll. N.S. Borchsenius.

#### Host plant.

Fagaceae: *Pasania* sp.

#### Distribution.

China (Yunnan).

#### Remarks.

After study of original material, *Lachnodiopsis szemaoensis* Borchsenius was transferred to *Paraputo* as *P. szemaoensis* (Borchsenius) by Williams (2004). As it has more than six setae on the anal ring, Danzig and Gavrilov-Zimin (2015) recognised *Lachnodiopsis* as a junior synonym of *Formicococcus* and made a new combination, *F. szemaoensis* (Borchsenius). Because the presence of more than six ring setae has been treated as an invalid generic character herein, we still agree with the opinion of Williams (2004) and consider *Lachnodiopsis* to be a junior synonym of *Paraputo*.

### Paraputo yunnanensis sp. n.

Taxon classificationAnimaliaHemipteraPseudococcidae

http://zoobank.org/45C76AB6-4D06-496F-B744-329B7D052F24

Material examined.

*Holotype*. Adult female. China: Yunnan, Lincang city, on *Eriobotrya japonica* (Rosaceae), xi.2015, coll. Yi-wei Fang. *Paratypes*. Five adult females, China: Yunan, Lincang city, on *Eriobotrya japonica* (Rosaceae), iii.2016, coll. Yi-wei Fang.

#### Description.

**Figure 3.**
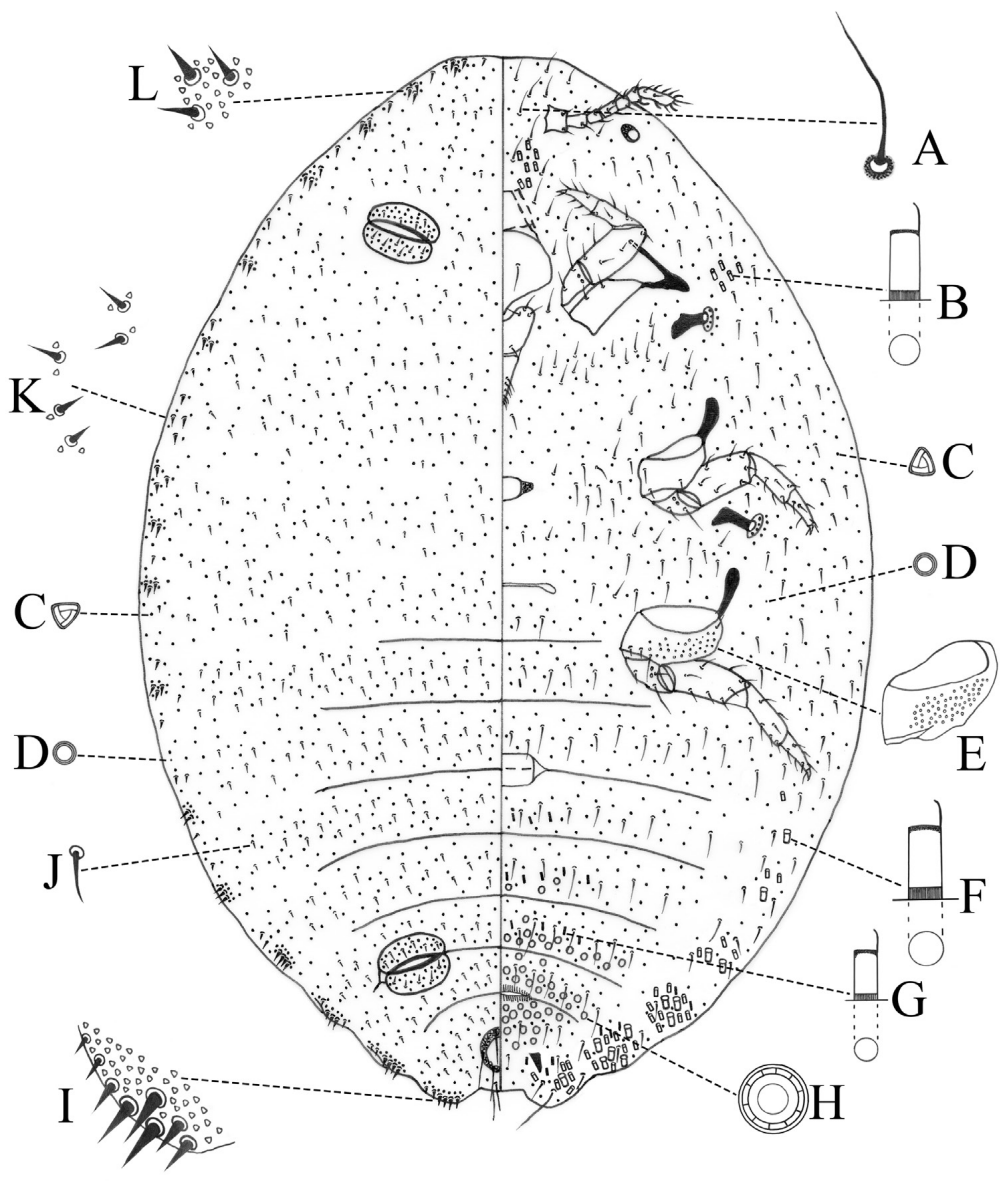
Adult female of *Paraputo yunnanensis* sp. n. **A** Ventral flagellate seta on head **B** Intermediate type of oral collar tubular duct **C** Trilocular pore **D** Discal pore **E** Hind coxa **F** Large type of oral collar tubular duct **G** Small type of oral collar tubular duct **H** Multilocular disc pore **I** Anal lobe cerarius **J** Dorsal seta **K** Cerarius on thorax **L** Cerarius on head.

Body of adult female on microscope slide (Fig. 3) broadly oval to rotund, 1.9–2.7 mm long and 1.4–1.9 mm wide. Anal lobes prominent, ventral surface of each lobe with small sclerotized area and apical seta 99–119 μm long; ratio of lengths of apical seta to anal ring seta 1: 0.68–0.86.

*Dorsum*. Ostioles well-developed, with inner edges of lips sclerotized, each lip with 4–7 short setae and numerous trilocular pores. Cerarii probably numbering 18 pairs, but thoracic cerarii usually poorly defined, with enlarged setae on thorax usually spaced far apart. Anal lobe cerarii (C_18_) each containing 6–12 conical setae of different sizes, large setae each 17–22 μm long and 7.5–9.5 μm wide at base, and a group of trilocular pores. Cerarii on posterior abdominal segments V–VII (C_15_–C_17_), each containing 4–7 conical setae; other cerarii, each containing two or three conical setae. Anal ring 87.5–92.5 μm long and 75–79 μm wide, situated nearly one times its length from apex of abdomen; bearing 6 short setae, each 70–85 μm long, slightly shorter than anal ring. Trilocular pores each 3.5–4 μm wide, numerous, evenly distributed. Multilocular disc pores and oral collar tubular ducts absent. Cisanal and obanal setae present, stout, each 50–79 μm long. Dorsal setae short, each 7.5–28 μm long. Setae flanking anal ring short, approximately same length as other dorsal setae. Discoidal pores, each smaller than a trilocucular pore, sparsely present.

*Venter*. Antennae each 270–347.5 μm long, 7-segmented; apical segment longest, bearing four fleshy setae. Eye prominent, located at body margin behind antennal base. Legs well developed, stout; hind coxa 77.5–95 μm long, hind trochanter + femur 237.5–280 μm long, hind tibia + tarsus 208.5–237.5 μm long; claw stout, 43.5–45 μm long, without denticle, claw digitules each knobbed and as long as claw. Ratio of lengths of hind tibia + tarsus to hind trochanter + femur 1: 1.14–1.2. Ratio of lengths of hind tibia to tarsus 1: 0.75–0.84. Translucent pores present on hind coxa. Clypeolabral shield 182.5–210 μm long. Labium 231.5–251.5 μm long. Ratio of lengths of labium to clypeolabral shield 1: 0.75–0.91. Circulus present, 90 μm long and 91.5–147.5 μm wide, situated between abdominal segments III and IV, divided by intersegmental line. Trilocular pores evenly distributed, fewer than those on dorsum. Multilocular disc pores, each 8–9 μm in diameter, present posterior to vulva and on abdominal segments VI and VII, few present on abdominal segment V, sometimes 1–4 present on each anal lobe. Oral collar tubular ducts of 3 sizes: large type, each 8–9.5 μm long and 4–5 μm wide, present in small marginal groups mainly on abdominal segments IV–VIII; intermediate type, each 8–9 μm long and 3.5–4 μm wide, present in marginal groups mainly on segments IV–VIII and in a group lateral to each anterior coxa, others present on frons, between antennal bases and clypeolabral shield; small type, each 5.5–6.5 μm long and 3 μm wide, distributed across abdominal segments IV–VI, some present on margin also. Ventral setae slender, longer than those on dorsum, each 19–45 μm long. Discoidal pores, same as those on dorsum, sparsely present.

#### Host plant.

Rosaceae: *Eriobotrya japonica*.

#### Distribution.

China (Yunnan).

#### Etymology.

The specific epithet is based on the name of the type locality Yunnan, combined with the Latin suffix ‘‘-ensis’’, indicating its place of origin.

#### Remarks.

*Paraputo yunnanensis* sp. n. is similar to *P. banzigeri* Williams in possessing oral collar tubular ducts on the head anterior to the clypeolabral shield, and in having the cerarii on the thorax sometimes indistinct. However, it differs from *P. banzigeri* Williams by the following features (condition of *P. banzigeri* Williams given in parentheses): (i) all the dorsal setae are short and pointed (dorsal setae on abdominal segment VIII much longer than other dorsal setae); (ii) translucent pores present only on hind coxa (pores not only present on hind coxa, but also on hind femur and hind tibia).

### Key to adult females of *Paraputo* species known from China

**Table:** 

1	Anal ring bearing six setae	**2**
–	Anal ring bearing multiple setae	**5**
2	Tubular ducts present between antennal bases and clypeolabral shield	**3**
–	Tubular ducts absent from between antennal bases and clypeolabral shield	**4**
3	Setae flanking anal ring noticeably longer than other dorsal setae; translucent pores present on hind coxa, hind femur and hind tibia	***P. banzigeri* Williams**
–	Setae flanking anal ring short, nearly same length as other dorsal setae; translucent pores present on hind coxa only	***P. yunnanensis* sp. n.**
4	Translucent pores present on hind coxa; venter of each anal lobe with sclerotized area	***P. albizzicola* Borchsenius**
–	Translucent pores absent from hind coxa; venter of each anal lobe membranous	***P. porosus* Borchsenius**
5	Cerarii numbering 5–7 pairs, present only on posterior abdominal segments	***P. szemaoensis* (Borchsenius)**
–	Cerarii numbering 11–18 pairs, present on head and thorax as well as on abdominal segments	**6**
6	Cerarii numbering 17 pairs; setae flanking anal ring short, approx. same length as other dorsal setae	***P. gasteris* Wang**
–	Cerarii numbering 18 pairs; setae flanking anal ring noticeably longer than other dorsal setae	**7**
7	Posterior three pairs of cerarii each situated on a sclerotized plate; translucent pores absent from hind coxa	***P. comantis* Wang**
–	Posterior three pairs of cerarii placed situated on membranous cuticle; translucent pores present on hind coxa	***P. platani* sp. n.**

## Supplementary Material

XML Treatment for Paraputo

XML Treatment for Paraputo albizzicola

XML Treatment for Paraputo banzigeri

XML Treatment for Paraputo comantis

XML Treatment for Paraputo gasteris

XML Treatment for Paraputo platani

XML Treatment for Paraputo porosus

XML Treatment for Paraputo szemaoensis

XML Treatment for Paraputo yunnanensis
